# Vertical Redistribution of Black Soldier Fly Larvae (*Hermetia illucens*) Under Restricted Airflow

**DOI:** 10.3390/insects17050531

**Published:** 2026-05-21

**Authors:** Shu-Wei Lin, Matan Shelomi

**Affiliations:** Department of Entomology, National Taiwan University, Taipei 106319, Taiwan

**Keywords:** aerotaxis, BSF, ethology, mass-rearing, ventilation

## Abstract

The black soldier fly, *Hermetia illucens* (Linnaeus, 1758), is a globally farmed insect reared to convert organic waste into useful biomatter and materials. While research on optimizing bioconversion rates and larval development through substrate manipulation has surged, studies on larval behavioral responses to abiotic factors lag. This benchtop-scale study is the first to examine larval behavior in response to reduced airflow. The results suggest third to fifth instar *H. illucens* larvae, which typically burrow within the substrate and avoid the surface, are significantly more likely to aggregate at the surface of their substrate when in a restricted airflow environment. More than half of third instar and nearly all 5th instar larvae aggregated at the surface when airflow was restricted, compared to less than a quarter under ventilated conditions. This data should stimulate future directions of hypothesis building in larval behavioral and physiological responses to airflow with the goal of using behavior modification to facilitate larval production.

## Simple Summary

The behavior of black soldier fly, *Hermetia illucens* (Linnaeus, 1758), larvae under restricted airflow conditions was unknown. This study tested how larvae arrange themselves vertically in a tube of substrate when the airflow is blocked by a lid. We hypothesized that larvae would move towards the surface rather than stay buried in the substrate, including earlier instars that typically remain buried. The results show that even 24 h of restricted airflow has a significantly strong effect, causing larvae to move upwards in a column, including early instar larvae that typically stay buried.

## 1. Introduction

The black soldier fly *Hermetia illucens* (Linnaeus, 1758) larvae (BSFL) are increasingly grown worldwide for their ability to bioconvert a wide range of wastes into usable biomatter [1,2]. The BSFL subsist on their substrate and rapidly digest it using hook-like mouthparts [3]. They feed collectively [4], crawling towards food on the surface from below, feeding, and being expelled from the top like a “fountain” [5]. BSFL are normally thigmotactic and negatively phototropic, preferring to congregate near the walls or bottom of a container away from the surface [6]. They normally show negative gravitropism, until they reach the sixth instar, a dark-colored “prepupa” stage in which they move to the substrate surface, exit, and “wander” away to find a different location to pupate [7].

Much contemporary research on this species has had an applied focus, such as optimizing larval performance by manipulating their dietary substrate’s nutrition [8,9], microbiology [10,11,12], and feeding system [13] to boost development and bioconversion efficiency. However, some basic, biological questions about *H. illucens* remain. BSFL behavioral responses to different rearing conditions, especially abiotic ones [13,14,15], are underexplored and often overlooked in studies focused on larval performance across various diets [16]. For example, high moisture content reduces larval survival [17] and compromises the effectiveness of larvae-residue separation via sifting machines [18,19,20], but its effects on larval locomotion and spatial distribution within a substrate along humidity gradients are unexplored.

The effects of aeration on larval locomotion and spatial distribution within a substrate are also understudied, be it the effects of ventilation of the rearing facility, airflow over the substrate, or the porosity of the substrate itself affecting oxygen and CO_2_ levels within [21]. Larvae typically feed in the top 2–4 cm of a substrate [22] but are capable of breathing at deeper depths, especially as aggregations of larvae in a substrate can improve oxygen penetration [23]. Manufacturers often provide deeper substrates and improve ventilation by churning, fanning [18,24], or forced gas release through tubes distributed in a substrate [25]. Aeration of the substrate with a fan improves larval development [16], final weight, and yield [25,26]. However, too much active aeration can dehydrate a substrate and increase larval mortality as the substrate dries to larvae and they become stuck on the sides of rearing containers [22,25]. Insufficient aeration in closed rearing chambers can lead to pasty substrates that also stick to the larvae and are harder to physically separate from larvae during harvesting [10]. Harvested BSFL can reportedly survive without oxygen when vacuum-packed for three to four days, though this is not considered a viable packaging method [27]. Outside of these studies that focus on applied metrics like yield, however, the effects of airflow on larval behavior itself are unknown.

The objective of this study was to investigate BSFL’s microenvironmental preferences in response to restricted airflow and to identify stimuli that may trigger larval vertical redistribution within a substrate [28]. The hypothesis underpinning this work was that restricting airflow in a substrate can trigger larvae to move to the surface, even those in the earlier instars that normally stay buried in the substrate.

## 2. Materials and Methods

*Hermetia illucens* eggs were sourced from an industrial supplier (Black Soldier Fly Fresh Insect Shop, Yutong Township, Yunlin, Taiwan). After hatching, the larvae were transferred to a larger container and fed *ad libitum* with a starter diet of waste soybean residue in a shaded, outdoor rearing facility until they reached the desired instar (3rd for early instar, 5th for late instar).

To examine the vertical stratification of larvae of different instars within substrates under conditions of restricted and unrestricted airflow, the following experiments were carried out. Three acrylic tubes (8.4 cm internal diameter, 4 cm height) were vertically stacked on a stainless steel plate, with the bottom tube sealed to the plate using modeling clay to ensure an airtight interface. Seams between tubes were sealed with parafilm, leaving the top opening as the sole air entry point. The inside would be filled to a depth of 9 cm with substrate. This construction, which can be taken apart quickly, was used to enable rapid harvesting of the larvae from three different depths: the surface and top 1 cm of substrate, 1–5 cm below the surface, and 5–9 cm below the surface.

The substrate used was a commercial chicken feed formulated for chicks (Formosa Oilseed Processing Co., Ltd., Taipei, Taiwan) made of corn and soy meal with a particle size range of 1.19–2.00 mm and ≤13% water. The substrate was mixed with dH_2_O to 54% water by weight before being used to fill the tubes. For each experimental trial, 50 larvae at the desired instar stage were randomly selected from the colony using soft forceps and deposited onto the substrate surface. The instars used were fifth instar, which have almost reached final size but have not yet darkened or begun the wandering characteristic of pre-pupae, or third instar larvae, with instars roughly distinguishable by size [29].

This study employed a 2 × 2 factorial design with larval instar (early/third instar vs. late/fifth instar) and airflow condition (mesh lid [unrestricted airflow] vs. acrylic lid [restricted airflow]) as fixed factors. Three replicates for each treatment combination were done, which is the same sample size used in previously published studies on BSFL and aeration [25] and other recent BSFL studies [30,31,32,33,34]. All setups were incubated in a growth chamber at 28 °C and 70% humidity with a 0:24 light–dark cycle for 24 h. After 24 h, each setup was removed from the growth chamber, the substrate was excavated carefully, and the number of larvae in each tube segment (top, middle, bottom) was recorded. Recall that the top tube contained 1 cm of substrate and 3 cm of space above the surface of the substrate.

For statistical analysis, because the experiment has a 2 × 2 factorial design with compositional count data, a multinomial generalized linear regression model with three responses (top, middle, and bottom tube) and two predictors (lid and instar) was run using the *VGAM* package of R. Post hoc comparisons were not performed using Tukey’s HSD because the response variable was multinomial rather than continuous. Instead, inference was based on likelihood ratio tests and model-based contrasts of predicted probabilities. A simple model was run testing each factor’s effects on larval distribution without interactions, which is statistically appropriate given the experimental design and sample size. The significance of factors within the model was tested with likelihood ratio chi-square tests comparing nested models.

## 3. Results

The mean percentages of larvae found in the bottom, middle, and top tubes of the treatment containers are given in Table 1. With mesh lids, most larvae were located in the middle tube at depths of 1–4 cm in the substrate, but with acrylic tubes, most were found in the top tube, either at a depth of 1 cm or entirely out of the substrate. This was the case regardless of instar, though late instar larvae were more likely to be at the top tube than early instar larvae.

Both lid and instar had significant (*p* < 0.001) impacts on these percentages according to chi-square testing of the multinomial generalized logistic regression model using the number in the bottom as the reference category. The multinomial generalized logistic regression model showed significant differences in larval distribution among sections (residual deviance = 42.79, df = 16). McFadden’s pseudo-R^2^ was calculated as 0.7466, which is considered an extremely strong fit, indicating strong explanatory power of instar and airflow condition on larval vertical distribution. The interaction between instar and airflow condition was not significant (χ^2^ = 4.9678, df = 2, *p* < 0.1) and was removed from the final model. Both instar (χ^2^ = 42.867, df = 2, *p* < 0.001) and airflow condition (χ^2^ = 290.04, df = 2, *p* < 0.001) had significant effects on larval vertical distribution. Using the bottom tube as the reference category in the model, late instar larvae were significantly more likely than early instar larvae to occupy the top section (β = 1.21, SE = 0.51, z = 2.39, *p* < 0.05), but not the middle section (*p* > 0.1). Acrylic lids that restricted airflow increased occupancy of the top section (β = 1.16, SE = 0.54, z = 2.16, *p* < 0.05) and decreased occupancy of the middle section (β = −1.88, SE = 0.50, z = −3.78, *p* < 0.001) relative to the bottom. Table 1 contains superscripted letters showcasing significant differences per row based on these results. These coefficients are in log-odds form, so the odds ratios are *e^* β. Ergo, BSFL in tubes with restricted airflow were 3.19 times as likely to be in the top and 0.15 times as likely to be in the middle relative to the bottom compared to BSFL in tubes with unrestricted airflow lids. Fifth instar larvae overall were 3.35 times as likely to be in the top, but not more or less likely to be in the middle, than third instar larvae.

## 4. Discussion

The main findings of this study were that larvae in tubes with restricted airflow (acrylic lid) were more likely to be at the surface or top layer of the container than larvae in tubes without restricted airflow, regardless of instar. Late instar larvae were also more likely to be at the top than third instar, regardless of airflow. Under the control condition of unrestricted airflow, larvae behaved as expected, aggregating within 1–5 cm of the substrate [22]. Under restricted airflow conditions, larvae moved towards or onto the surface, with, on average, over 90% of fifth instar larvae showing this behavior.

This is the first work that establishes BSFL behavioral responses to restricted airflow conditions. This work has some clear limitations, notably in its very small scale compared to industrial rearing or even household-scale BSFL reactors. Nonetheless, the results suggest that reducing airflow, even for 24 h, triggered larvae to congregate at the top of the substrate, if not on the surface and out of the substrate altogether, in contrast to their normal behavior of burying themselves inside it [22]. This result is especially noteworthy in that it also applies to earlier instar larvae that lack the negative gravitropism of prepupae.

This vertical redistribution as a result of restricted airflow can have multiple causes [22], none of which were directly tested in this study or by any prior studies. These could include gradients in O_2_/CO_2_ levels [23], humidity [35], temperature [23], microbial volatiles [23], and ammonia levels [18] that form or change in intensity with reduced or increased airflow to a BSFL substrate; however, data from the literature on these is severely lacking. No studies to date have tested any of these possible correlates of oxygen gradients with BSFL behavior before, so future experimentation is needed to test these effects separately under more controlled conditions by measuring microenvironmental factors at different depths. The larger percentage of fifth instar larvae at the top compared to third instar suggests those top-layer individuals may have been preparing to molt to prepupae, escape the substrate, and wander, though there is no prior data on relative vertical distribution of BSFL during these final larval moments to validate this hypothesis. Other topics for future research include BSFL oxygen requirements, mobility and locomotion through substrates of different porosities and permeabilities to oxygen and water, and their degrees and intensity of aerotaxis, hygrotaxis, thermotaxis, etc.

The ability to cause larvae to leave a substrate and aggregate at the surface can have several practical applications, particularly for small-scale reactors. While current industrial-level BSFL production systems typically take all larvae and residue and separate them using large sieves [20,36,37], a medium-sized, closed-reactor system could theoretically employ reduced ventilation in this manner to harvest larvae without disturbing or discarding the waste or to harvest larvae at a stage earlier than the wandering pre-pupa should that be desirable. Methods using larval behavior to facilitate harvesting have been proposed before [28], but none using airflow limitation. Other potential uses of brief airflow restriction to bring larvae to the surface include a quick, non-invasive check of larval density and activity or a means of sampling larvae for research or inspection purposes without removing the substrate. Finally, should a producer note the presence of larvae at the substrate surface unintentionally, that could be an indicator of anaerobic conditions or insufficient airflow and a sign to ventilate the substrate.

## Figures and Tables

**Table 1:** Percentage of n = 50 larvae by count (mean across three replicates ± standard deviation) in the top, middle, and bottom tubes of the test chambers relative to the chamber lid and larval instar. Different superscript letters indicate the values within each row are significantly different based on the post hoc comparisons on the model predictors from the *VGAM* package in R.

	Mesh Lid		Acrylic Lid	
	3rd Instar	5th Instar	3rd Instar	5th Instar
Top (≥1 cm)	10.00 ± 4.00 ^d^	23.30 ± 8.33 ^c^	64.40 ± 8.72 ^b^	92.00 ± 10.58 ^a^
Middle (4 cm)	85.33 ± 1.16 ^a^	72.00 ± 5.29 ^a^	26.20 ± 3.46 ^b^	4.00 ± 5.29 ^b^
Bottom (4 cm)	4.67 ± 4.16 ^a^	4.70 ± 4.16 ^a^	9.40 ± 8.08 ^a^	4.00 ± 5.29 ^a^

## Data Availability

All relevant data is provided in this publication.

## Abbreviations

The following abbreviations are used in this manuscript:

BSFL	Black Soldier Fly Larvae
